# Quality of primary care delivery and productive interactions among community-living frail older persons and their general practitioners and practice nurses

**DOI:** 10.1186/s12913-019-4255-2

**Published:** 2019-07-16

**Authors:** Lotte Vestjens, Jane M. Cramm, Anna P. Nieboer

**Affiliations:** Erasmus School of Health Policy and Management, P.O. Box 1738, Rotterdam, 3000 DR The Netherlands

**Keywords:** Quality of primary care, Productive patient-professional interactions, Chronic care model, Elderly, Frailty

## Abstract

**Background:**

Although there is evidence with respect to the effectiveness of Chronic Care Model (CCM)-based programs in terms of improved patient outcomes, less attention has been given to the effect of high-quality care on productivity of patient-professional interactions, especially among frail older persons. The aim of our study was therefore to examine whether frail community-dwelling older persons’ perspectives on quality of primary care according to the dimensions of the CCM are associated with the productivity of the patient-professional interactions.

**Methods:**

Our study was part of a large-scale evaluation study with a matched quasi-experimental design to compare outcomes of frail community-dwelling older persons that participated in a proactive, integrated primary care approach based on (elements of) the CCM and those that received usual primary care. Frail older persons’ perceptions of quality of care were assessed with the Patient Assessment of Chronic Illness Care Short version (PACIC-S). Productive interactions with general practitioners (GPs) and practice nurses were assessed using a relational coproduction instrument. Measurements were performed at baseline (T0) and 12 months thereafter (T1). In total, 232 frail older persons were participating in the intervention group at T0 and matched to 232 frail older persons in the control group. At T1, 182 persons were in the intervention group and 176 in the control group.

**Results:**

Paired sample *t*-tests showed significant improvements in overall quality of care, the majority of underlying quality of care items, and productive interactions within the intervention group and control group over time. Multilevel analyses revealed that productive interaction with the GP and practice nurse at T1 was significantly related to perceived productive interaction with them at T0, the perceived quality of primary care at T0, and the change in perceived quality of primary care over time (between T0 and T1).

**Conclusions:**

Frail community-dwelling older persons’ perspectives on quality of primary care were associated with perceived productivity of their interactions with the GP and practice nurse in both the intervention group and the control group. We found no significant differences in overall perceived quality of care and perceived patient-professional interaction between the intervention group and control group at baseline and follow-up. In times of population aging it is necessary to invest in high-quality care delivery for frail older persons and productive interactions with them.

## Background

Providing essential components of high-quality, proactive, integrated primary care and support for frail community-dwelling older persons is a great challenge for current healthcare systems [1–3]. Redesign of the primary healthcare system is inevitable to facilitate the provision of high-quality, proactive, integrated care, which requires comprehensive and complex transformations in a primary care setting [4]. The Chronic Care Model (CCM) proposes important system changes that can guide primary care practices to improve quality of care and patients’ outcomes [4–7]. Organizational change is proposed in six key areas: delivery system design, self-management support, decision support, clinical information systems, the healthcare system, and the community [4, 6, 8].

The premise of the CCM is that system changes are considered essential in fostering productive interactions between (teams of) healthcare professionals and their patients and, ultimately, improve patient outcomes [9]. These productive patient-professional interactions should emphasize shared decision-making and partnerships between professionals and patients to produce the best possible outcomes [10]. Productive patient-professional interactions involve reciprocal interrelating between healthcare professionals and patients [11–13] and can be thought of as a mutually reinforcing cycle of communicational and relational aspects [14]. Improved patient outcomes are more likely to be achieved when the patient and the healthcare professionals communicate effectively (frequent, timely, accurate, and problem-solving communication). Effective communication is reinforced by the nature of relationships between patients and healthcare professionals. These relationships include shared goals, shared knowledge, and mutual respect. In turn, these relational dimensions are reinforced by frequent, timely, accurate, and problem-solving communication between patients and professionals [12, 14, 15]. Productive interactions are accomplished by, for example, providing systematic assessments, supporting patients’ self-management abilities, optimizing treatments, and providing sufficient follow-up. A necessity for interactions to be productive is that patients are activated and informed actors in their care process. Therefore, patients need to have relevant information, sufficient skills, and confidence to be involved in decision-making processes. Besides the changing roles of older patients in the care process, the roles of healthcare professionals also need to change. High-quality care is characterized by proactive and prepared (teams of) healthcare professionals that have the necessary expertise, patient information, time, and resources to conduct productive interactions and to ensure effective care coordination [4]. Productive interactions between activated and informed patients and prepared and proactive healthcare professionals are at the heart of patient-centered care delivery. Patients’ preferences and needs should be respected, patients should be engaged in the decision-making process, and care should be tailored to optimize patient outcomes [16]. Especially in the care for older persons with often long-term complex (healthcare) needs and (multiple) chronic conditions productive patient-professional interactions seem to be important. Holman and Lorig [17] underline that chronic illness care compared with acute care practices necessitates patients to be active partners in managing their health and (chronic) illnesses. This requires a continuous process in which the person contributes and participates at almost all levels of decision-making and action taking [17].

### The CCM and productive interactions – previous research

Although there is evidence with respect to the effectiveness of CCM-based programs in terms of, for example, improved quality of care delivery and patient outcomes [18], less attention has been given to the effect of high-quality care on the productivity of the patient-professional interactions. According to Cramm and Nieboer [19], evidence that care based on (elements of) the CCM leads to productive patient-professional interactions is limited [19]. Productive interaction is an essential element of person centered care [16] and considered important for improving patient outcomes [4, 5, 9].

Research has shown that (changes in) the quality of care as perceived by professionals and chronically ill patients enhanced productivity of interactions [19, 20]. Productive patient-professional interactions, in turn, are associated with improved outcomes like well-being among patients with (multiple) chronic conditions [21, 22]. Besides, the relationship between patients’ perceptions of quality of care and their well-being was mediated by productive interactions. Therefore, with respect to improving patient outcomes, it is important to establish productive interactions between healthcare professionals and patients and to invest in high-quality integrated care delivery [21]. To our knowledge, however, in a population of community-dwelling frail older persons evidence with respect to the relationship between perceived quality of care delivery that is in line with (elements of) the CCM and productivity of patient-professional interactions is lacking. Care approaches based on (elements of) the CCM have primarily focused on patients with specific chronic conditions [18, 23]. There are only few comprehensive approaches aimed at delivering integrated (healthcare) services according to elements of the CCM in (frail) older persons in a primary care setting [24–26]. In times of population aging, research into the perceived quality of CCM-based care approaches and the relationship with the productivity of patient-professional interactions in a population of community-dwelling frail older persons is crucial.

### Study aim

The aim of our study was to examine whether frail community-dwelling older persons’ perspectives on quality of primary care according to the dimensions of the CCM are associated with the productivity of the interactions with the general practitioner (GP) and practice nurse. We aimed to comparatively assess quality of care and productive patient-professional interactions in a population of frail older persons receiving a proactive, integrated primary care approach called *Finding and Follow-up of Frail older persons (FFF approach)* and frail older persons receiving usual primary care in the Netherlands.

## Methods

### Context of the study: primary care in the Netherlands

According to Erler and colleagues [27], primary care is considered ‘the spine’ of the healthcare system in the Netherlands [27]. Dutch primary healthcare organization is strong compared with primary healthcare in many European countries [28]. Dutch primary care includes a broad range of services and (health) professions (e.g., physiotherapists, and pharmacists). Central to the primary care system are GP practices and GPs with a gatekeeping function. Hospital and specialist care are in most cases only accessible upon referral [29, 30]. Almost all Dutch citizens are registered with a GP, mainly in the persons’ living area. Patients consult their GP generally on their own initiative. Appointments are commonly planned within 2 days and general practice care is excluded from the mandatory deductible associated with the obligatory basic health insurance. In the Netherlands, GPs are commonly non-interventionist, with low prescription and referral rates to secondary care [29]. Over the years, the primary care setting and the division of labor among primary care professionals has been reformed. GPs increasingly work in teams and larger organizations, like group practices. Task delegation and differentiation is occurring and as a result other professions such as practice nurses are working in GP practices [29–31]. GPs and practice nurses are the most frequently consulted healthcare providers in primary care [32]. One important pillar of the reforms in the long-term care over the past years is the transition from institutional care to care in the home-setting [33]. Care for older persons with often complex (healthcare) needs and multiple chronic conditions is increasingly organized in the primary care setting [34, 35]. As a result, the complexity of patient care in the primary care setting is increasing [35, 36]. Fragmentation between primary healthcare and other sectors is still predominant and has been considered an important barrier in enhancing coordination and continuity in the care for persons with complex (healthcare) needs [37–39]. Traditionally, primary care is largely focused on providing reactive and curative care and focuses less on proactive and preventive care [27, 36].

### Study design

The current study is part of a large-scale evaluation study with a matched quasi-experimental design to compare outcomes of frail community-dwelling older persons that participated in the proactive, integrated primary care approach FFF (intervention group) and those that received usual primary care (control group). The study was conducted in the western part of the Province of North Brabant in the Netherlands between 2014 and 2017. We approached 17 GP practices for participation in the evaluation study (12 intervention GP practices and 5 control GP practices). In total, 1 intervention practice and 1 control practice were not willing to participate due to the workload and time constraints. The intervention group consisted of frail community-living older persons of 11 GP practices providing care and support according to the FFF approach. The control group consisted of frail independently living older persons of 4 GP practices providing care as usual. Participating GP practices varied in practice size, practice location (urban or rural locations; although the distance to other healthcare facilities remained limited), solo or duo/group practices and (number of) disciplines in the practice (e.g., practice nurses). Measurements were performed at baseline (T0) and at 12 months thereafter (T1). The research proposal has been reviewed by the medical ethics committee of the Erasmus Medical Centre in Rotterdam, the Netherlands (study protocol number MEC-2014-444). The committee decided that the rules laid down in the Medical Research Involving Human Subjects Act (Dutch acronym: WMO) did not apply. Consequently, further examination for ethics approval was waived by the medical ethics committee. Written informed consent to participate in the study was obtained from all participants. The assignment of the intervention was not under the discretion of the investigators, consequently registration of our study as a trial was not required. More details of the study design have been published elsewhere [40].

### Participants and inclusion

The study population consisted of frail independently living older persons in the age of 75 years and older. With increasing age, the prevalence of frailty increases [41, 42]. We used a four-stepped approach to assess frailty, include older persons in the FFF approach and in the evaluation study, and perform one-to-one matching. *Step 1:* We assessed frailty among all community-dwelling older persons registered at the 15 participating GP practices, i.e. 4 control GP practices and 11 intervention GP practices. All older persons received a postal questionnaire, including the 15-item Tilburg Frailty Indicator (TFI), which was developed and validated by Gobbens and colleagues [43]. The TFI is used to assess frailty in the physical, psychological, and social domains [43]. The TFI is based on the definition of frailty as proposed by Gobbens and colleagues [44], that is ‘Frailty is a dynamic state affecting an individual who experiences losses in one or more domains of human functioning (physical, psychological, social), which is caused by the influence of a range of variables and which increases the risk of adverse outcomes’ [44]. Respondents with a TFI score of 5 or higher (range 0–15) were identified as frail [43]. The aim of this frailty assessment was twofold, namely (i) to assess frailty in community-living older persons, and (ii) to attain frailty scores for the matching procedure (Step 4). *Step 2:* Frailty scores were provided to the participating GPs in order to give them insight into the proportion of frail older persons in their GP practice. *Step 3:* GPs in intervention GP practices selected older persons that were included in the FFF approach. This selection could be guided by older persons’ frailty scores and/or additional interviews or measures that were performed by healthcare professionals as part of their care delivery. *Step 4:* The persons that were included in the FFF approach were assessed on inclusion criteria for participation in the evaluation study by the researchers. Frail older persons eligible for inclusion in the evaluation study (i) were living independently in the community, (ii) did not have an estimated life expectancy of less than 3 months, and (iii) were able to communicate in Dutch. The researchers matched each older person in the intervention group to one older person in the control group on key covariables, namely sex (male or female), score on the TFI, and educational level (low or high). Quasi-experimental research designs are more susceptible to bias due to the absence of randomization [45, 46]. One-to-one matching was performed to increase the comparability of the intervention and control groups.

**Fig. 1:**
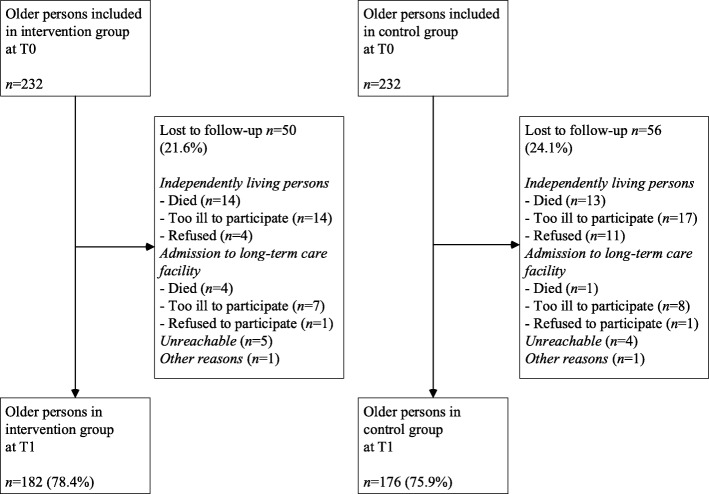
Flowchart of study participation

Written informed consent was obtained from all included older persons before the baseline data collection. As illustrated by Fig. 1, 232 frail older persons were participating in the intervention group at T0 and matched to 232 frail older persons in the control group. At T1, 182 older persons were in the intervention group and 176 in the control group (loss to follow-up rates of 21.6 and 24.1% respectively).

### Intervention and control groups

Community-dwelling older persons in the intervention group (11 GP practices) received the proactive, integrated primary care approach FFF (for a description of the approach see paragraph ‘Intervention: steps in the FFF approach’). In the control group (4 GP practices), frail older persons received usual care services as provided by GP practices and other healthcare and community organizations. The control GP practices were instructed not to implement (elements of) the FFF approach. See paragraph ‘Context of the study: primary care in the Netherlands’ for more information about the Dutch primary healthcare system.

### Intervention: steps in the FFF approach

**Fig. 2:**
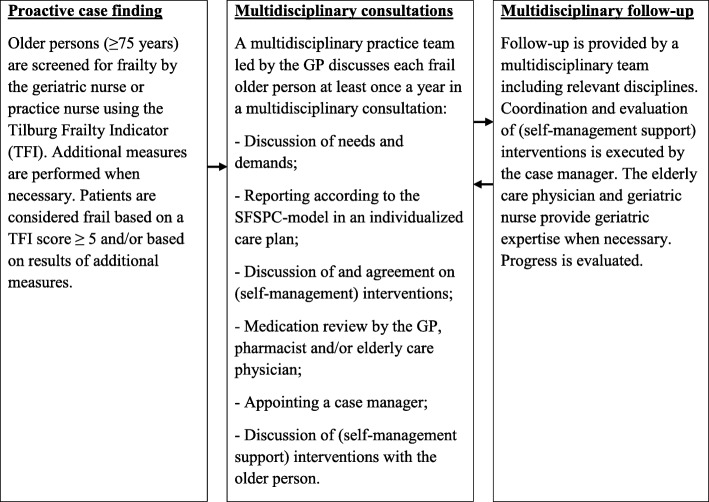
Overview of phases in the proactive, integrated FFF approach

The FFF approach is aimed at providing high-quality proactive, integrated primary care for community-dwelling frail older persons (75 years and older) in order to meet their often complex and long-term (healthcare) needs and protect their well-being. The FFF approach consists of several steps (see Fig. 2). First, community-living older persons aged 75 years and older are screened for frailty by means of the TFI [43] by the geriatric nurse or practice nurse during a home visit. Additional measures are performed when necessary (e.g., Mini–Mental State Examination (MMSE)). The results of the home visit are discussed with the elderly care physician, i.e., primary care expert in geriatric medicine and care for older patients with complex needs [47, 48]. The results are reported and submitted to the GP and serve as input for the multidisciplinary consultation. Second, each frail older person is discussed in multidisciplinary consultation. The multidisciplinary practice team includes preferably the practice nurse, geriatric nurse, elderly care physician, and is led by the GP. This practice team can be strengthened by other disciplines, like physiotherapists or professionals in social care. Each frail older person is discussed in multidisciplinary consultation at least once a year. Needs and demands of the frail older person are discussed and reported according to the SFSPC-model (Somatic, Functional, Social, Psychological, and Communicative indications) in an individualized care plan. The practice team discusses and agrees upon (self-management) interventions. Over-the-counter and prescribed medicines are examined in a medication review by the GP, pharmacist and/or elderly care physician. Additional actions can be introduced, like coordinating medication use between primary care and second-line medical care, and a consult of the elderly care physician to provide specific information about medicines to the frail older person. A case manager is appointed for each frail older person. The discipline that is most frequently involved in the care and support for the older person takes up the coordination role in the care process. The individualized care plan including proposed (self-management) interventions is discussed with the frail older patient and adjusted to the person’s needs and wishes. Finally, follow-up of the frail older person is provided by a multidisciplinary team involving disciplines relevant for the (healthcare) needs and demands of the frail older person, e.g., GP, elderly care physician, physiotherapist, geriatric nurse, and social worker. The case manager coordinates and evaluates the effectiveness of the executed (self-management) interventions (during home visits) at least every 3 months. The elderly care physician and the geriatric nurse work in tandem to provide specialized geriatric expertise in the follow-up of frail older patients. The GP can obtain advice about, for example, multimorbidity, dementia, depression, and falls. Progress is evaluated and discussed in multidisciplinary consultation. Additional interventions or disciplines are introduced when necessary (see Fig. 2).

### The FFF approach and the CCM

The FFF approach was based on (elements of) the CCM. The comprehensive FFF approach combines multifaceted interventions related to changes in the *delivery system design* that enable effective care delivery, such as implementing case management, and working in multidisciplinary teams. Several *self-management support* interventions are provided, like providing educational interventions, personal coaching, and individualized care plans and goals. The integrated care approach provides *decision support* for healthcare professionals by implementing guidelines for geriatric care in the primary care setting and professional training for care providers, and aims to enhance *clinical information systems* (e.g., facilitate exchange of information among care disciplines). The *healthcare system* promotes care improvement and strategies at multiple organizational levels (e.g., financing geriatric care modules and agreements with health insurers) and supports regional collaboration with *community* organizations. In line with the premises of the CCM, the FFF approach aims to improve quality of primary care, foster productive patient-professional interactions, and subsequently influence frail older persons’ well-being.

### Data collection and measures

Quality of primary care, productive patient-professional interactions and background characteristics of frail older persons were assessed by means of interviews (i.e. administering questionnaires) at home by trained interviewers at T0 and T1 (12 months follow-up). Interviewers were kept blinded to the group the older person was in (intervention or control group). On average, interviews lasted 60 to 75 min.

#### Measuring perceptions of quality of primary care

Frail older persons’ perceptions of quality of primary care delivery were assessed with the 11-item Patient Assessment of Chronic Illness Care Short version (PACIC-S), which was validated by Cramm and Nieboer [49]. The PACIC-S was based on the 20-item Patient Assessment of Chronic Illness Care (PACIC) as developed and validated by Glasgow and colleagues [50]. The PACIC assesses patients’ perspectives on care delivery according to the dimensions of the CCM [50]. Frail older persons were asked to indicate the extent to which they received CCM-related actions and care on a five-point scale ranging from ‘(almost) never’ to ‘(nearly) always.’ Higher scores represent higher-quality primary care delivery as perceived by frail older persons. Cronbach’s alpha values for the PACIC-S were 0.77 at T0 and 0.76 at T1.

#### Measuring productive interactions with the GP and practice nurse

Productive patient-professional interactions were assessed using a relational coproduction instrument which measures dimensions of communication and relationships [51–54]. We focused specifically on productive interactions with the GP and the practice nurse. The relational coproduction instrument contains seven survey questions assessing frequency, timeliness, accuracy, and problem-solving nature of communication and the quality of the relationships in terms of shared goals, shared knowledge, and mutual respect. These dimensions of communication and relationships constitute jointly the relational coproduction construct [51–54]. Example questions are: ‘Do these professionals communicate accurately with you?’ and ‘Do these professionals share the same goals as you?’ Frail independently living older persons assessed the quality of the communication and relationships with their GP and practice nurse on a five-point scale ranging from ‘never’ to ‘always.’ Higher scores represent higher-quality productive patient-professional interactions as perceived by frail older persons. Cronbach’s alpha values for the productive patient-professional interactions were good (with the GP 0.86 at T0 and 0.88 at T1, and with the practice nurse 0.89 at T0 and 0.84 at T1).

### Statistical analyses

The study population at baseline was described by means of descriptive statistics. Independent samples *t*-tests and chi-squared tests were used to assess baseline differences. Paired sample *t*-tests were used to investigate differences in scores at T0 and T1 on individual PACIC-S items, mean overall PACIC-S scores, and mean overall scores on relational coproduction within the intervention group and control group. Independent samples *t*-tests were used to assess differences between groups in mean PACIC-S scores and mean scores on relational coproduction at T0 and T1. Linear mixed-effects models were used to investigate the relationships between (changes in) frail older persons’ perceptions of quality of primary care and productivity of interactions with the GP and practice nurse. Multilevel models are considered appropriate for investigating relationships in data sets with continuous dependent variables and a clustered structure of the data [55]. A random intercept was used on the level of the individual GP practices. Outcome estimates in the multilevel analyses were adjusted for baseline values of the outcome variables, background variables (i.e., age, sex, marital status, educational level, and multimorbidity) and the group (control and intervention group) was included in the model. Results were considered statistically significant when two-sided *p*-values were < 0.05. We used software package IBM SPSS for Windows (version 24) for all statistical analyses.

## Results

### Baseline characteristics

Table 1 shows the baseline characteristics of the older persons at baseline. At baseline, older persons in the intervention group were significantly less often single compared with older persons in the control group. No significant differences between the intervention group and control group were found with respect to age, sex, educational level, frailty or multimorbidity.

**Table 1:** Baseline characteristics of older persons

	Intervention group *n* = 232	Control group *n* = 232
Age (years)	82.45 (5.44)	82.41 (5.16)
Sex (female)	168 (72.4%)	168 (72.4%)
Marital status (single)	134 (57.8%)	160 (69.0%)*
Educational level (low)	101 (43.5%)	91 (39.2%)
Frailty (score on TFI)	7.38 (2.40)	7.38 (2.39)
Multimorbidity (≥ 2 diseases)	214 (92.6%)	206 (89.6%)

Values are presented as mean (SD, standard deviation) or number (%)

Independent samples *t*-tests and Chi-squared tests. * *p* < 0.05 (two-tailed)

Note: Characteristics of the population were based on the baseline measurement T0

### Perceived quality of primary care

Table 2 shows the mean quality of primary care delivery scores as measured with the PACIC-S (mean overall scores) as well as mean scores on individual PACIC-S items for frail independently living older persons in the intervention group and control group. Paired sample *t*-tests showed significant improvements in the mean overall PACIC-S score within the intervention group over time and within the control group over time. We found significant improvements in mean scores on 9 out of 11 individual PACIC-S items over time in the intervention group as well as in the control group. Improvements were seen in the following quality of care items: ‘given choices on treatment to think about’, ‘satisfied that my care was well organized’, ‘helped to set specific goals to improve my eating or exercise’, ‘encouraged to go to a specific group/class to help me cope with my (chronic) illness’, ‘asked questions about my health habits’, ‘helped to make a treatment plan that I could do in my daily life’, ‘helped to plan ahead so I could take care of my illness even in hard times’, ‘asked how my (chronic) illness affects my life’ and ‘told how my visits with other (healthcare) professionals helped my treatment’. No significant differences in mean scores on items ‘given a copy of my treatment plan’ and ‘contacted after a visit of the GP, nurse or medical specialist to see how things were going’ were found in both the intervention group and control group between T0 and T1. Moreover, independent samples *t*-tests showed no significant differences in mean overall scores of the PACIC-S between the control and intervention groups at T0 (1.83 (SD = 0.61) vs. 1.84 (SD = 0.56); *p* = 0.80) and at T1 (2.25 (SD = 0.69) vs. 2.31 (SD = 0.63); *p* = 0.38).

**Table 2:** Quality of primary care as experienced by frail older persons in the intervention and control groups over time (T0 and T1) based on paired data

	Intervention group *n* = 149^b^		Control group *n* = 144^c^	
Item characteristics of the PACIC-S^a^	T0	T1	T0	T1
Given choices on treatment to think about	2.25 (1.36)	2.89 (1.56)***	2.11 (1.39)	2.99 (1.73)***
Satisfied that my care was well organized	4.22 (1.11)	4.52 (0.93)**	4.17 (1.25)	4.52 (1.04)**
Helped to set specific goals to improve my eating or exercise	1.74 (1.09)	2.57 (1.49)***	1.52 (0.99)	2.27 (1.51)***
Given a copy of my treatment plan	1.44 (1.08)	1.36 (0.75)	1.35 (0.94)	1.49 (1.11)
Encouraged to go to a specific group/class to help me cope with my (chronic) illness	1.28 (0.76)	1.70 (0.76)***	1.20 (0.63)	1.56 (1.04)**
Asked questions about my health habits	1.98 (1.30)	2.34 (1.34)**	1.74 (1.26)	2.29 (1.48)***
Helped to make a treatment plan that I could do in my daily life	1.40 (0.89)	1.85 (1.03)***	1.38 (0.91)	1.66 (0.99)*
Helped to plan ahead so I could take care of my illness even in hard times	1.39 (0.85)	1.91 (1.11)***	1.41 (0.94)	1.66 (0.99)*
Asked how my (chronic) illness affects my life	1.53 (1.07)	1.79 (1.03)*	1.51 (1.11)	1.78 (1.15)*
Contacted after a visit of the GP, nurse or medical specialist to see how things were going	1.81 (1.27)	1.81 (1.18)	1.59 (1.14)	1.66 (1.13)
Told how my visits with other (healthcare) professionals helped my treatment	1.83 (1.29)	2.81 (1.47)***	1.62 (1.13)	2.73 (1.64)***
Mean overall score of the PACIC-S^a^				
Perceived quality of primary care	1.90 (0.56)	2.32 (0.63)***	1.78 (0.54)	2.24 (0.70)***

Values are presented as mean (SD, standard deviation)

Paired sample *t*-tests. *p < 0.05 (two-tailed); **p < 0.01 (two-tailed); *** *p* < 0.001 (two-tailed)

^a^PACIC-S, Patient Assessment of Chronic Illness Care Short version, range 1–5; ^b^149 persons of the 182 older persons included in the intervention group at T1 completed both measurements (T0 and T1) for the PACIC-S, 0–1 missing per item of the PACIC-S; ^c^144 persons of the 176 older persons included in the control group at T1 completed both measurements (T0 and T1) for the PACIC-S, 0–1 missing per item of the PACIC-S

### Perceived productive interactions with the GP and practice nurse

Table 3 shows frail older persons’ perceptions of productive interactions with their GP and practice nurse (mean scores on the relational coproduction instrument). Paired sample *t*-tests showed significant improvements in perceived productive interactions with the GP in the intervention group over time and within the control group over time. We also found significant improvements with respect to frail older patients’ perceived productive interactions with the practice nurse in the intervention group over time and within the control group over time. Moreover, independent samples *t*-tests showed no significant differences between the control and intervention groups with respect to productive interactions with the GP and practice nurse at T0 (3.77 (SD = 1.19) vs. 3.80 (SD = 1.11); *p* = 0.75, and 2.44 (SD = 1.69) vs. 2.64 (SD = 1.68); *p* = 0.21 respectively) and at T1 (4.45 (SD = 0.85) vs. 4.33 (SD = 1.04); *p* = 0.23, and 3.86 (SD = 1.70) vs. 3.77 (SD = 1.68); *p* = 0.61 respectively).

**Table 3:** Perceived productive interaction with the GP and practice nurse in the intervention group and control group over time (T0 and T1) based on paired data

Perceived productive interactions^a^	*n*	T0	T1
Intervention group			
Productive interaction with the GP	172	3.90 (1.03)	4.35 (1.01)***
Productive interaction with the practice nurse	172	2.84 (1.70)	3.76 (1.68)***
Control group			
Productive interaction with the GP	165	3.78 (1.19)	4.45 (0.86)***
Productive interaction with the practice nurse	164	2.45 (1.67)	3.87 (1.69)***

Values are presented as mean (SD, standard deviation)

Paired sample *t*-tests. *** *p* < 0.001 (two-tailed)

^a^Relational coproduction instrument, range 1–5

### Determinants of productive interactions with the GP and practice nurse

Tables 4 and 5 show the results of the multilevel analyses. Productive interaction with the GP at T1 was significantly related to the perceived productive interaction with the GP at T0, the perceived quality of primary care at T0, and the change in perceived quality of primary care over time (between T0 and T1). There was no significant relationship with background characteristics and the group the frail older patient was in, i.e. intervention group or control group (Table 4). Analyses showed that the perceived productive interaction with the practice nurse at T0, the perceived quality of primary care at T0, and the change in perceived quality of primary care over time were significantly related to productive interaction with the practice nurse at T1. Also, we found no significant relationship with background characteristics and the group the patient was in (Table 5).

**Table 4:** Determinants of productive interactions with the GP at T1 as assessed with multilevel analysis (*n* = 292)

	B	SE
Constant	2.64**	0.85
Intervention group	−0.12	0.09
Perceived productive interaction with GP T0	0.15**	0.04
Perceived quality of primary care T0	0.37**	0.11
Change in perceived quality of primary care (T1 – T0)	0.37***	0.07
Age	0.01	0.01
Sex (female)	−0.18	0.11
Marital status (single)	0.08	0.10
Educational level (low)	0.02	0.10
Multimorbidity	−0.23	0.18

*SE* standard error; Multilevel analyses included respondents that filled in the questionnaires at both T0 and T1. Deletion of missing cases resulted in 292 cases

**p < 0.01 (two-tailed); ***p < 0.001 (two-tailed)

**Table 5:** Determinants of productive interactions with the practice nurse at T1 as assessed with multilevel analysis (*n* = 291)

	B	SE
Constant	3.04	1.72
Intervention group	−0.31	0.27
Perceived productive interaction with practice nurse T0	0.15*	0.06
Perceived quality of primary care T0	0.46*	0.22
Change in perceived quality of primary care (T1 – T0)	0.45**	0.14
Age	0.001	0.02
Sex (female)	−0.23	0.22
Marital status (single)	0.18	0.21
Educational level (low)	0.06	0.20
Multimorbidity	−0.28	0.37

*SE* standard error; Multilevel analyses included respondents that filled in the questionnaires at both T0 and T1. Deletion of missing cases resulted in 291 cases

*p < 0.05 (two-tailed); **p < 0.01 (two-tailed)

## Discussion

The aim of our study was to investigate whether frail community-dwelling older persons’ perspectives on quality of primary care according to the dimensions of the CCM are associated with perceived productivity of interactions with their GP and practice nurse. We have found significant improvements in perceived care quality, perceived productive interaction with the GP, and perceived productive interaction with the practice nurse over time in both the intervention group (proactive, integrated primary care) and the control group (usual care delivery). There were no significant differences between the intervention group and control group with regard to overall perceived quality of primary care and perceived interactions with the GP and practice nurse at baseline and at follow-up. Productive interactions with the GP and practice nurse were significantly related to the perceived productive interaction at T0, the perceived quality of primary care at T0, and the change in perceived quality of primary care over time (between T0 and T1). The quality of the communication and relationships between frail community-dwelling older persons and their GPs and practice nurses is associated with the perceived quality of primary care delivery.

The rich history of the central position of the GP in primary care may explain why on average community-living frail older persons scored the productivity of interactions with their GP higher compared with the interactions they encounter with the practice nurse, a relatively newer professional within GP practices. Still the perceived productive interaction with the practice nurse was scored relatively high in our sample of frail community-dwelling older persons. A study among COPD patients has shown the highest degree of productivity of interactions with the nurse practitioner and GP compared with other professionals such as specialists [20].

Earlier research has shown that care delivery in accordance with the CCM is associated with productive patient-professional interactions as perceived by chronically ill patients [19, 20]. In addition, productive patient-professional interactions mediated the relationship between care quality as perceived by chronically ill patients and their well-being [21]. Our study adds to this knowledge by showing that perceived quality of primary care is associated with perceived productive patient-professional interaction in a sample of frail community-living older persons, which is expected to influence their well-being as well. This stresses the necessity to invest in high-quality care delivery and interactions between frail older patients and their healthcare professionals.

The outcomes should be viewed in the light of the setting in which we conducted our study. The effectiveness of the FFF approach in improving quality of primary care and productive patient-professional interactions may depend on the organization of the healthcare setting. Considering the strongly developed Dutch primary care system, the contrasts between proactive, integrated care as provided in the FFF approach and usual care delivery might not have been large enough. Based on a comparison of the results of three studies investigating integrated care programs for community-dwelling frail older persons in the Netherlands [24, 56, 57], Hoogendijk [58] states that integrated care adds little to the usual care delivery in the Dutch primary care setting. Jackson, Scott, and Gutierrez [59] state that the effects of integrated healthcare would be greater in healthcare systems that are more fragmented, like the healthcare system in the United States.

We have found suboptimal implementation of elements related to the FFF approach in intervention GP practices. For example, GP practices differed in their organization of multidisciplinary consultations (e.g., how often consultations were organized, number of older persons discussed, which type of (healthcare) professionals were involved) and the way they arranged long-term follow-up of frail older persons. Moreover, during the study period initiatives to improve care delivery for older persons were also reported in the control GP practices. Even though these practices did not deliver care and support according to the FFF approach, systematic follow-up of older patients, implementing chain information systems, creating a structural approach between hospital and primary care, and the delegation of care from GP to the (practice) nurse are examples of changes that also took place in several control GP practices [60]. Quality improvement initiatives and possibly other trends in primary care for older persons may have contributed to improvements over time. For a detailed description of implemented interventions in intervention and control GP practices see Vestjens, Cramm, and Nieboer [60].

### Limitations of the study

The study has several (potential) limitations. First, we measured frail community-dwelling older persons’ perceived productivity of interactions with their GP and practice nurse. We decided to limit our selection of professionals to the GP and practice nurse, which are the most frequently contacted professionals in general practice in the Netherlands [32]. We experienced problems with measuring productive interactions with other healthcare professionals that were part of the practice team supporting frail older patients, such as elderly care physicians and social workers. In general, it was difficult for participants to recognize the disciplines that were less visible to them than their GP or practice nurse which made it complicated to successfully investigate older patients’ perceived productive interactions with these professionals. The productivity of interactions with other healthcare professionals requires therefore further investigation. This is important as multidisciplinary teamwork is an important element of the proactive, integrated care approach FFF. Second, the control GP practices that agreed to join may already have had high-levels of quality of care and may have been highly motivated to improve the quality of their care delivery. These GP practices may have perceived that the FFF program would add no value to their usual care delivery, and subsequently may have been particularly eager to participate in the control group. Healthcare practices with medium or low levels of quality of care delivery may decline requests to participate in evaluation studies whereas those who are doing well may be more likely to join. This may hamper our ability to detect changes between intervention practices and care as usual. Third, based on the theoretical underpinnings of the CCM [4, 6, 8], we investigated the relationships between (changes in) care quality and productive patient-professional interactions. This relationship, however, may be considered dynamic. Higher-quality productive interactions are expected to result in higher-quality primary care for frail older persons (e.g., improved self-management support) [54]. Furthermore, we did not include other potential predictors of (the relationship between) care quality and productive interactions. For example, continuity of care is found to be an important predictor of high quality of primary care [61]. In the current study we did not take into account the duration and/or intensity of patient care provided by the GP or practice nurse. Although we applied matching methods and controlled for important factors in the data analyses, this provides no guarantee for unbiased results. Other unknown and unmeasured factors to confound our study results may exist. Finally, our study focused on older persons’ perceptions of quality of primary care and productive patient-professional interactions only. We did not investigate whether improvements resulted in improved patient outcomes, like health-related quality of life or well-being of community-dwelling frail older persons. The effects on patient outcomes should be investigated in future research.

## Conclusions

The aim of the study was to investigate whether frail community-dwelling older persons’ perspectives on quality of primary care are associated with the productivity of patient-professional interactions. Frail community-dwelling older persons’ perspectives on quality of primary care were associated with perceived productivity of their interactions with the GP and practice nurse in both the intervention group receiving proactive, integrated care based on (elements of) the CCM and the control group receiving care as usual. We found no significant differences in overall perceived quality of care and perceived patient-professional interaction between the intervention group and control group at baseline and follow-up. Our study contributes to previous research by showing that perceived quality of primary care is associated with perceived productive patient-professional interaction among frail community-dwelling older persons. In general, less research has been conducted with respect to the relationship between quality of care and productivity of patient-professional interactions, while effective interactions are assumed to positively influence patient outcomes. In times of population aging it is therefore necessary to invest in high-quality care delivery and patient-professional interactions. The effects of improvements in quality of primary care and productive patient-professional interactions on patient outcomes of frail community-dwelling older persons need to be examined in future research.

## Data Availability

The datasets generated and analyzed during the current study are not publicly available, but are available from the corresponding author on reasonable request.

## Abbreviations

CCM: Chronic Care Model
FFF: Finding and Follow-up of Frail older persons
GP: General practitioner
MMSE: Mini–Mental State Examination
PACIC(−S): Patient Assessment of Chronic Illness Care (Short version)
SD: Standard deviation
SE: Standard error
SFSPC: Somatic, Functional, Social, Psychological, and Communicative indications
TFI: Tilburg Frailty Indicator
